# *I3*: A Self-organising Learning Workflow for Intuitive Integrative Interpretation of Complex Genetic Data

**DOI:** 10.1016/j.gpb.2018.10.006

**Published:** 2019-11-23

**Authors:** Yun Tan, Lulu Jiang, Kankan Wang, Hai Fang

**Affiliations:** 1 State Key Laboratory of Medical Genomics and Shanghai Institute of Hematology, Ruijin Hospital, Shanghai Jiao Tong University School of Medicine, Shanghai 200025, China; 2 Bristol Renal, School of Clinical Sciences, University of Bristol, Bristol BS1 3NY, UK; 3 Wellcome Centre for Human Genetics, University of Oxford, Oxford OX3 7BN, UK

**Keywords:** Self-organising, Human genetics, Interpretation, Evolution, Machine learning

## Abstract

We propose a computational workflow (*I3*) for intuitive integrative **interpretation** of complex genetic data mainly building on the **self-organising** principle. We illustrate the use in interpreting genetics of gene expression and understanding genetic regulators of protein phenotypes, particularly in conjunction with information from human population genetics and/or evolutionary history of human genes. We reveal that loss-of-function intolerant genes tend to be depleted of tissue-sharing genetics of gene expression in brains, and if highly expressed, have broad effects on the protein phenotypes studied. We suggest that this workflow presents a general solution to the challenge of complex genetic data interpretation. *I3* is available at http://suprahex.r-forge.r-project.org/I3.html.

## Introduction

We know the exciting promise in machine learning applied to genetics and genomics [1]. We also know to date there has been relatively slow progress achieved by machine learning, in terms of how to intuitively make sense of emerging genetic datasets. Now we are able to generate many new types of genetic datasets, for example, through genetic mapping of gene expression across tissues [2] and genetic screens for protein phenotype regulators [3], [4]. However, our ability to understand such datasets is very limited. The rate of data interpretation is much slower in particular when seeking to integrate with population-wide genetic information and species-wide evolutionary information. Population-wide genetic variants could be aggregated into a metric estimating the loss-of-function intolerance of a gene [5], while evolutionary history of human genes could be estimated by phylostratigraphy [6], [7] defining evolutionary age for a gene as our ancestor in which this gene was first appeared. Data interpretation should be also made considering well-annotated knowledge on genes, usually in the form of signaling pathways such as from Reactome [8]. One of the challenges is how to integrate all information accelerating interpretation, ideally achieved in a single workflow.

**Figure 1:**
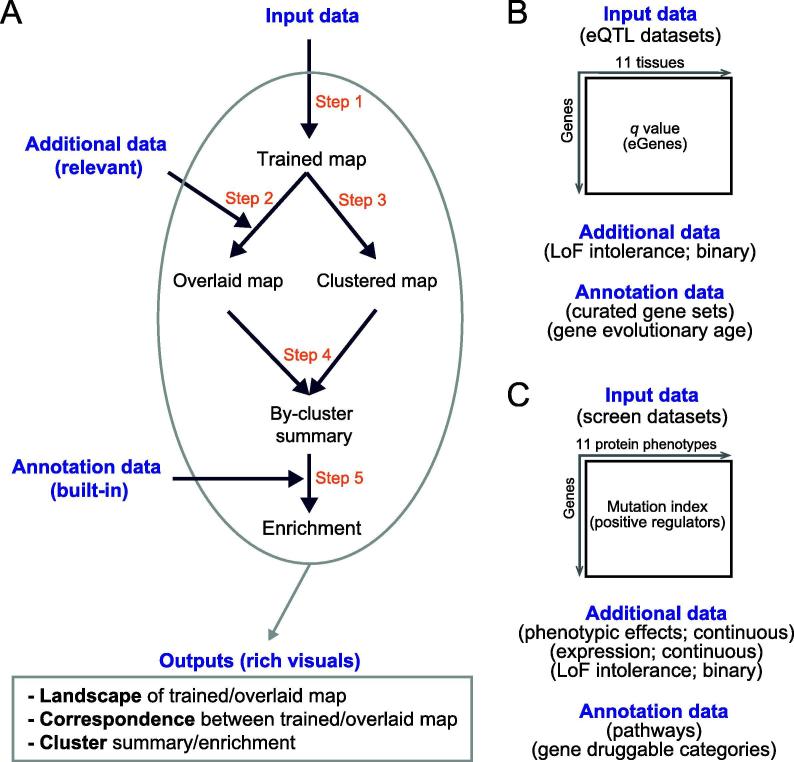
**Overview of *I3* enabling intuitive integrative interpretation** **A.***I3* workflow. **B.** Applications to the interpretation of eQTL genes (eGenes) in brain tissues in terms of additional data (LoF intolerance) and annotation data (curated gene sets and gene evolutionary ages). **C.** Applications to the interpretation of genetic regulators in terms of additional data (protein phenotypic effects, gene expression, and LoF intolerance) and annotation data (pathways and gene druggable categories). eQTL, expression quantitative trait loci; LoF, loss-of-function.

To address the challenge above, we propose a computational workflow that enables characterisation of input data and integration with additional (relevant) data for knowledge discovery, all achieved in an intuitive way (Figure 1A). This workflow benefits from three considerations. Firstly, we characterise input data using a self-organising learning algorithm [9]. This may be most applicable for its unsupervised nature. As comparisons, supervised machine learning (such as deep learning [10]) requires desired outcomes as part of learning that are usually not available for computational biology. Secondly, the self-organising ability is desirable for unbiased integration with additional datasets that can be diverse in data types (binary and continuous). Thirdly, data characterisation is constrained on a regularly shaped map. This is no trivial as the regular map is much easier for effective visualisations. Because of these considerations, one of the defining features in our workflow is the map-centric interpretation that covers all steps of interpretations (overlaying/integration, clustering, enrichment, and other downstream analyses that are scalable to meet customised needs).

Our workflow was inspired by the previous work, that is, implementing the self-organising principle to interpret regulatory genomics [11], gene expression patterns [12], [13], accessible chromatin [14], and DNA replication timing [15], to name but just a few. To further advocate this principle and also to demonstrate the value and applications of the proposed workflow, we interpreted two complex genetic datasets: one generated from multi-tissue expression quantitative trait loci (eQTL) mapping [2] (Figure 1B), and the other from haploid mutagenesis screens for protein phenotypes [3], [4] (Figure 1C). We view this workflow as Intuitive Integrative Interpretation or ‘*I3*’ because it mimics how we human beings, at our disposal, gather together the knowledge available best explaining the data.

## Methods

### Detailed description of the *I3* workflow

#### Step 1: self-organising input data constrained by the map shape

We extended a self-organising algorithm to map shapes trained from input data, made available as part of an R/Bioconductor package ‘supraHex’ [15]. The design of a map shape considers the structure of input data; principle component analysis (PCA) helps to reveal what data point clouds look like (either the shape boundary or the number of density centers). We visualised the trained map as the landscape in 1D or 2D. The support for the 1D landscape was simply done by visualising the codebook matrix associated with the trained map. For example, the trained tissue map was visualised providing a tissue-specific view of all its eGenes, collectively forming tissue landscape. The support for the 2D landscape was achieved by using a 2D square map lattice to self-organise, for example, protein phenotypes, in a way that geometric location within this 2D lattice delineates the similarity between them.

#### Step 2: obtaining the overlaid map by overlaying additional data onto the trained map

The algorithm used for overlaying was described previously [15] but based on newly designed map shapes. The trained map overlaid with an additional (non-training) data resulted in an overlaid map that is associated with an overlaid codebook matrix. As described at Step 1, this overlaid codebook matrix was used for landscape visualisation. The correspondence between input data and additional data was measured as Pearson’s correlation coefficient using the codebook matrix associated with the trained/overlaid map.

#### Step 3: identification of gene clusters from the trained map

We generalised a region-growing algorithm [15] to partition the trained map into gene clusters, each of which is continuous over the map.

#### Step 4: by-cluster summary of the overlaid map

The summary was calculated based on the overlap map (obtained at Step 3) by averaging values over each continuous cluster (identified at Step 2).

#### Step 5: enrichment analysis of identified clusters

The enrichment analysis was based on fisher’s exact test. This type of analysis can be conveniently renamed according to the knowledge used. Based on fisher’s exact test (two-tails), we performed curated gene set analysis and evolutionary analysis to identify both enrichments and depletions for gene clusters. We curated gene sets, including the developmental disorder genes from Developmental Disorders Genotype-to-Phenotype (DDG2P; 1724 genes mapped to EntrezGene; the same hereinafter) [16], ExAC LoF intolerance genes (3160 genes) [5], genes reported in the genome-wide association study (GWAS) Catalogue (5122 genes) [17], phenotype genes annotated using human phenotype ontology (HPO; 3522 genes) [18], and Online Mendelian Inheritance in Man (OMIM) disease genes (4212 genes) [19]. Evolutionary analysis for these gene clusters was based on 16 phylostrata, each representing a group of genes that appeared at a specific ancestor [6]. These phylostrata are ordered by the evolutionary history: Cellular organisms (1715 genes), Eukaryota (4525 genes), Opisthokonta (276 genes), Metazoa (1912 genes), Eumetazoa (1152 genes), Bilateria (1090 genes), Chordata (308 genes), Euteleostomi (2693 genes), Amniota (532 genes), Mammalia (512 genes), Theria (580 genes), Eutheria (731 genes), Euarchontoglires (119 genes), Catarrhini (211 genes), Homininae (252 genes), and Homo sapiens (25 genes). Based on fisher’s exact test (on-tail), we performed pathway analysis and druggable analysis to identify enrichments only. Pathway analysis was performed using Reactome pathways [8], and druggable analysis using DGIdb druggable gene categories [20].

### Datasets from human embryos, GTEx and haploid mutagenesis screens

We obtained human embryo transcriptome datasets involving 5441 differentially expressed genes/probesets and 6 successive developmental stages with three replicates for each stage [13]. We obtained 7890 eGenes (*q* value < 0.05) in brain subregions and the whole blood from GTEx (version 6p) [2]. Positive regulators for 11 protein phenotypes (FDR < 0.05; 1321 genes in total) were obtained according to studies using a random mutagenesis-based haploid screen [3], [4]. All these datasets were used as input data for training.

### Definition of loss-of-function (LoF) intolerant genes

We obtained LoF intolerant genes from the Exome Aggregation Consortium [5], defined as genes having at least 90% probability of LoF intolerance. This resulted in a status vector involving 17,568 genes, with 1 for LoF intolerance and 0 otherwise. This vector was used as additional data for overlaying.

### Phenotypic effects and expression levels of regulators

For each regulator identified by a random mutagenesis-based haploid screen [3], [4], we defined phenotypic effects as the number of phenotypes that this regulator was declared significant (FDR < 0.05). Its expression level was calculated as median of RNA-seq data of 10 independent wild-type HAP1 cells [3]. These continuous values were used as additional data for overlaying.

## Results and discussion

### Overview of intuitive integrative interpretation (*I3*)

*I3* is designed as a general and flexible workflow (Figure 1A) enabling map-centric intuitive interpretation of input data, allowing for integration with additional (relevant) data and knowledge discovery with annotation (built-in) data. As a general workflow, it can be used to interpret any input data (a numeric matrix containing, for example, genes in rows and measures in columns). As a flexible workflow, it can integrate any relevant additional data (also provided by the user) and provides built-in annotation data (such as evolution and pathways) for knowledge discovery. *I3* outputs rich visuals for intuitive interpretation, including landscape visualisation, correspondence between input and additional data, and identification of clusters and enrichments.

At the core of *I3* is the self-organising learning. In the literature, a number of tools have been reported for similar purposes, including *SOM Toolbox* [21], *Cluster 3.0* [22], and two R packages (*kohonen* [23] and *supraHex* [15]). Amongst these, *SOM Toolbox* is widely used but requires the commercial license (MATLAB). *Cluster 3.0* supports the graphical user interface but suffers from output visualisation. Both packages *kohonen* and *supraHex* are open source and similar in the use and visualisation. A major limitation of current tools is that all of them are limited in the choice of map shapes. All but *supraHex* supports the sheet-like map only. This is essential for modeling input data of diverse or unknown shapes. To illustrate this point, we used human embryo transcriptome datasets [13] and compared the trained map of different shapes. This data involves six successive developmental stages. We already know there are two groups of genes, displaying gradually reduced or gradually increased expression patterns; such *priori* knowledge can be used to assess the performance. PCA revealed two highly dense regions/centres of genes; for this we devised a butterfly-like map (Figure S1A). In doing so, we found that two groups of genes were nicely separated and mapped to each of two wings (Figure S1B). As comparisons, we also modeled the same data based on the sheet map and found that the separation boundary is less clear (Figure S1C).

*I3* consists of five steps (Figure 1A): training the map using the input data in a self-organising manner but constrained by the map shape (Step 1), obtaining the overlaid map by overlaying additional data onto the trained map (Step 2), identification of gene clusters from the trained map (Step 3), the by-cluster summary of the overlaid map (Step 4), and enrichment analysis of identified clusters using annotation data (Step 5). In this study, without loss of generality we applied the *I3* workflow to interpret two complex genetic datasets. Figure 1B gives a summary of data used to interpret eQTL genes (eGenes) in brain tissues mainly regarding LoF intolerant genes and genes at different evolutionary ages, while Figure 1C interprets genetic regulators mainly regarding protein phenotypic effects, gene expression and LoF intolerance.

### Interpreting genetics of gene expression in brains

**Figure 2:**
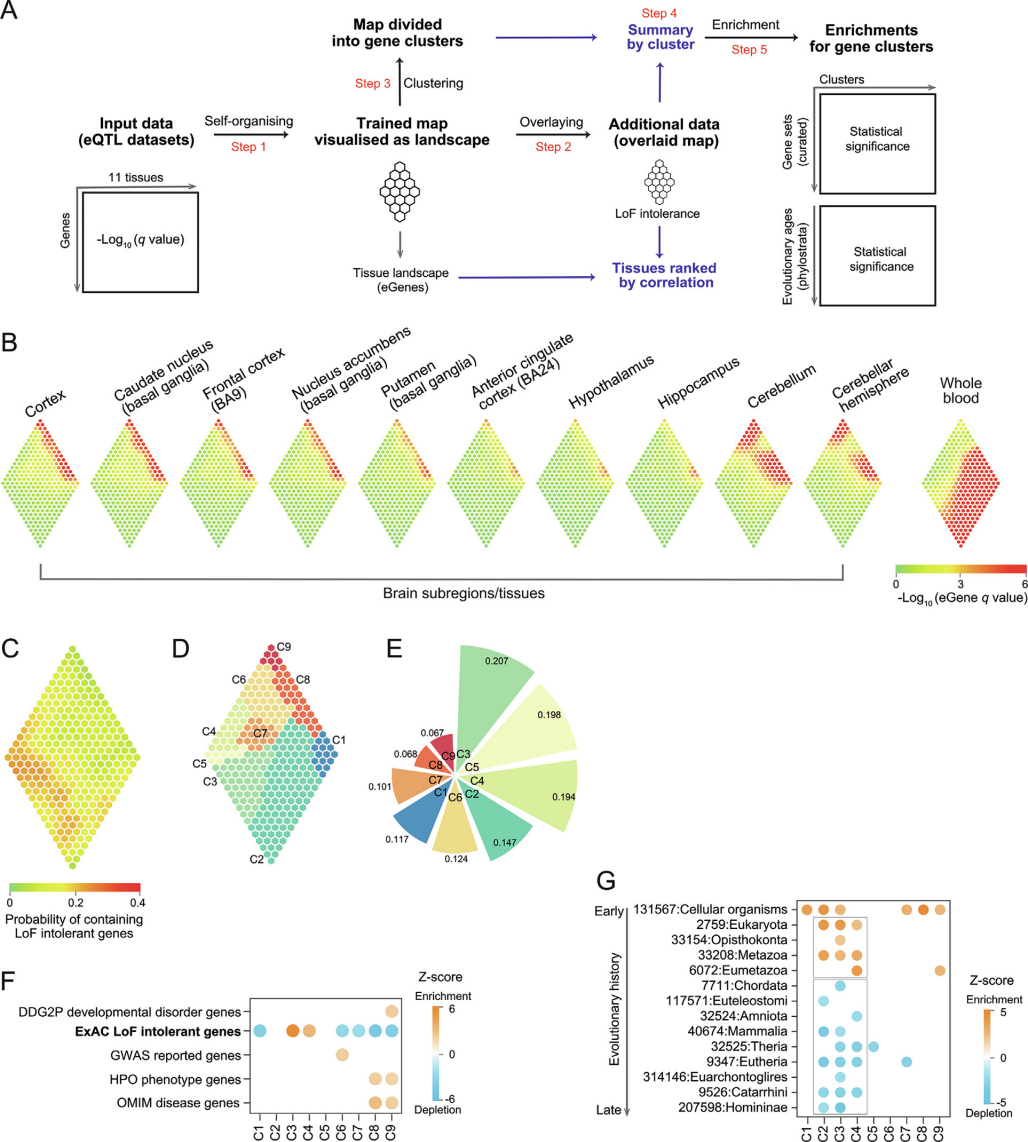
**Genetics of gene expression in brain tissues** **A.** Overview of analytical workflow. **B.** Brain tissue landscape. A diamond-shape map trained using GTEx eGene datasets in brain tissues (and the whole blood for comparisons). The colour bar represents the *q* value significance defining an eGene in a tissue. **C.** LoF intolerance map produced from the LoF intolerance data overlaid onto the trained tissue map. **D.** Gene cluster identified from the trained tissue map. Clusters color-coded and labeled. **E.** The probability of containing LoF intolerant genes averaged per cluster. **F.** Enrichment analysis of gene clusters (in columns) in terms of 5 curated gene sets (in rows). **G.** Evolutionary analysis of gene clusters. Shown in rows are phylostrata ordered by evolutionary history.

The Genotype-Tissue Expression (GTEx) project identified genetic variants associated with expression of genes (eGenes) in a tissue-specific manner [2]. Here we illustrate the power of *I3* in interpreting eGenes found in 10 brain subregions (and the whole blood as comparisons) with respect to their selective pressure against mutations (Figure 2A). An eGene for a tissue was defined if its expression is significantly regulated by a variant, measured by the q value. A gene under selective pressure was defined if extremely intolerant to LoF mutations.

#### A diamond-shaped map models tissue-specific eGenes in brains

We prepared the input data matrix with an eGene (in rows) found in a tissue (in columns), in which we observed a diamond-like shape of distribution (Figure S2A). Based on this, we designed a diamond-shape map and trained it by the input data via the self-organising learning algorithm. We visualised the trained map as the tissue landscape (Figure 2B), identifying the similar maps between brain tissues that show sharp difference as compared to the one seen in the whole blood. We also designed a triangle-shape map for comparisons and observed the more constrained distribution for eGenes, suggesting that this shape is less effective in completely unfolding input data (Figure S2C).

#### The trained tissue map is overlaid to produce the LoF intolerance map (Figure 2C)

By comparing the tissue landscape and the LoF intolerance map, we found that all brain tissues negatively correlated with LoF intolerance; this autocorrelation is much stronger than that seen in the whole blood (Figure S2B).

#### The trained tissue map is divided into gene clusters for knowledge discovery

We obtained a total of 9 gene clusters (C1–C9) from the tissue landscape (Table S1), each covering continuous regions (Figure 2D) and summarised by the probability of containing LoF intolerant genes (Figure 2E). We observed that tissue-specific eGenes in C3–C5 had a high probability of containing LoF intolerant genes, and a low probability for tissue-sharing eGenes in C8–C9. Enrichment analysis using curated gene sets confirmed this observation; we found that C3–C4 significantly enriched for LoF intolerant genes and C8–C9 significantly depleted of LoF intolerant genes (Figure 2F). We also observed enrichment for phenotype genes and disease genes in C8–C9, and developmental disorder genes enriched in C9 only. Notably, C9 contains eGenes shared by all brain tissues (not in the whole blood), least under selective pressure. To fortify the above findings, we also performed evolutionary age analysis. We found a preference of genes in C2–C3 to be created at our ancestor Eumetazoa or earlier, and these genes are unlikely to be created at our ancestor Chordata or later (Figure 2G). By contrast, we did not observe such evolutionary origin preference for genes in C7–C9. Based on the trained map in brains, we produced the map for other tissues (Figure S3A). For the clusters C8–C9 mostly depleted of LoF intolerant genes, we found the majority of tissues have eGenes. We also revealed that tissues (such as subcutaneous adipose, tibial artery, transformed fibroblasts, muscular esophagus, lung, skeletal muscle, tibial nerve, skin and thyroid) had a much higher number of eGenes. Collectively, *I3* reveals that LoF intolerant genes are depleted of tissue-sharing genetics of expression (not just in brains but most of other tissues; such relationship is more consistent for brain-derived tissues), and there exists a preference in their evolutionary origin. Without selective pressure in population and/or in evolution seems to be prerequisite for a gene causing diseased phenotypes and even developmental disorders.

### Integrative interpretation of genetic regulators of protein phenotypes

**Figure 3:**
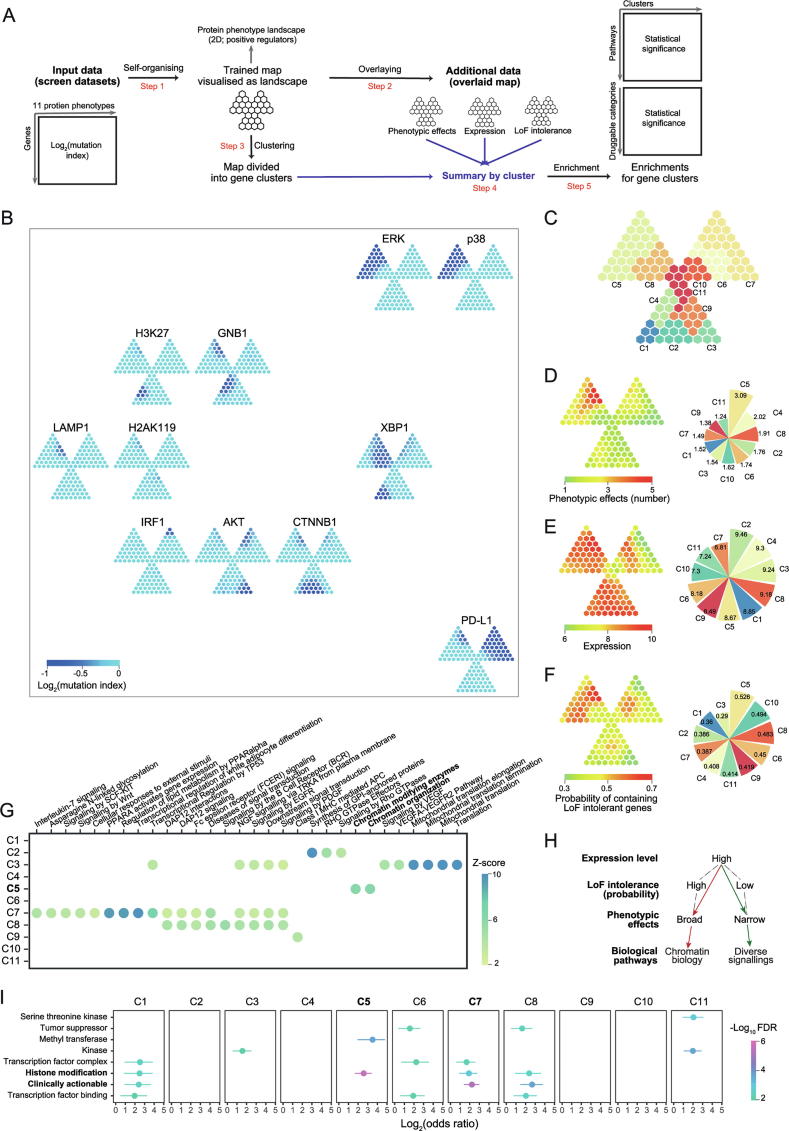
**Genetic regulators of protein phenotypes** **A.** Overview of analytical workflow. **B.** Protein phenotype landscape on 2D. A trefoil-shaped map trained from positive regulators involving 11 protein phenotypes. The colour bar represents the mutation index as a measure of identifying regulators; the lower the more likely, according to haploid mutagenesis screens for genetic regulators. The landscape is drawn within the outermost box in which geometric location depicts the similarity between these 11 protein phenotypes. **C.** Gene cluster identified from the trained protein phenotype map. Clusters color-coded and labeled. The overlaid map obtained by overlaying additional data onto the trained protein phenotype map. The phenotypic effect map using the per regulator number of phenotypes (**D**), the expression map using the RNA-seq expression data in HAP1 cells (**E**), and the ExAC LoF map using the ExAC LoF intolerance data (**F**). Also shown on the right are values for the corresponding additional data averaged per cluster. **G.** Reactome pathways enriched in gene clusters. **H.** LoF intolerance explaining relationships between expression and phenotypic effects of genetic regulators. **I.** Druggable categories enriched in gene clusters. Odds ratio (and 95% confidence interval) based on Fisher’s exact test. ATK, Phosphorylated ATK; CTNNB1, Non-phosphorylated β-catenin; ERK, Phosphorylated ERK; GNB1, GNB1 abundance; H2AK119, Histone H2A(K119) crotonylation; H3K27, Histone H3(K27) trimethylation; IRF1, IRF1 abundance; LAMP1, Glycosylated LAMP1; p38, Phosphorylated p38; PD-L1, PD-L1 abundance; XBP1, Spliced XBP1.

Haploid genetic screens are new techniques identifying genetic regulators of protein phenotypes by introducing random mutagenesis into haploid cells and linking such mutations to protein states as phenotypic readouts [3], [4]. We here demonstrate the utility of *I3* in understanding positive genetic regulators with respect to three factors: phenotypic effects, expression and LoF intolerance based on a trefoil-shape map (Figure 3A and Figure S4). The 2D landscape of protein phenotypes reveals information on both phenotypes and regulators (Figure 3B). Phenotypes with the similar regulator profile (*e.g.*, phosphorylated ERK and p38) are placed together, and far away for phenotypes with a very different profile (*i.e.*, PD-L1). Phenotype-sharing regulators are mostly mapped onto the top-left leaf of the trefoil, more precisely, gene cluster 5 (C5 in Figure 3C; Table S2). Indeed, regulators in C5 had broadest phenotypic effects (Figure 3D). The previous study showed that genes with high expression levels tend to be genetic regulators of protein phenotypes [3]. To further explain this, we examined the relationship between expression levels and phenotypic effects. We observed that regulators with high expression (Figure 3E) could have both broad phenotypic effects (C5) and narrow effects (C1–C4 and C8). With *I3*, we found that regulators in C5 had higher probability of containing LoF intolerant genes than those seen in C1–C4 and C8 (Figure 3F). We also found enrichment of chromatin biology in C5 (Figure 3G); this is consistent with the fact that ERK and p38 (important players in MAPK cascades) have broad regulatory impacts in gene expression and thus strong regulation on chromatin-related genes [24]. Interestingly, pathways (*e.g.*, signaling by EGFR) relevant to MAPK were not enriched in C5, implying the more complex genetic regulation involving these pathways than previously thought in the classical ‘EGFR-EGF-RAS-RAF-MEK-ERK’ axis. For clusters C2–C3 and C8, enrichment of diverse signallings was observed (Figure 3G). Taken together, *I3* reveals a putative model, that is, the LoF intolerance may act as a latent factor explaining the relationship between expression levels and phenotypic effects (Figure 3H). Beyond interpretation, we also explore the pharmaceutical use of genes identified by genetic screens, that is, to evaluate the druggability for each gene cluster. The top druggable gene category is ‘histone modification’ enriched in C5 (*BAZ1B*, *DOT1L*, *EED*, *EZH2*, *HCFC1*, *ING5*, *KAT7*, *KDM2A*, *KMT2A*, *MECP2*, *PHF8*, *PRMT1*, *SETDB1*, *SIN3B*, *SUZ12*, and *USP7*), followed by ‘clinically actionable’ genes (*APC*, *BAP1*, *BCL2L1*, *CD274*, *CREBBP*, *EWSR1*, *FBXW7*, *FLCN*, *GREM1*, *IFNGR1*, *JAK1*, *JAK2*, *KDM5C*, *NCOR1*, *NF2*, *NSD1*, *RB1*, *TSC1*, and *TSC2*) in C7, a gene cluster unique to PD-L1 (Figure 3I and Table S3). Given the broad phenotypic effects and high expression level in C5, we suggest those genes involved in histone modification should be given a high priority for follow-up in experiments.

## Conclusion

The *I3* workflow is implemented in the R running environment, an open source platform that is widely used, and thus can reach the wide audience. The workflow is designed in a non-linear and intuitive way (Figure 1) with the focus on the flexibility rather than the easy-to-use interface; this is one of current limitations that should be overcome in the future, for example, by developing a web server to remove the dependency on R. Another future effort is to automate the selection of the map shape or to explore other ways (beyond PCA) doing so; for example, in a less visual-aided way fine-tuning the specific parameters. Nonetheless, we have demonstrated the value of this workflow. Interpreting genetics of gene expression reveals a lack of selective pressure for tissue-sharing eGenes in brains. Interpreting genetic regulators of protein phenotypes points to the importance of LoF intolerance in bridging expression levels and phenotypic effects. Both applications are achieved in relatively short runtime (the training finished in seconds using one core on Mac OS X). To conclude, *I3* provides an integrated solution to complex genetic datasets for downstream interpretation and knowledge discovery.

## Availability

*I3* is available at http://suprahex.r-forge.r-project.org/I3.html.

## Authors’ contributions

YT performed the analysis and revised the manuscript. LJ revised the manuscript. KW conceived the project, contributed to the interpretation and edited the manuscript. HF conceived the project, performed the analysis and drafted the manuscript. All authors read and approved the final manuscript.

## Competing interests

The authors have declared no competing interests.
