# Supplementary figures and images for: I3: A Self-organising Learning Workflow for Intuitive Integrative Interpretation of Complex Genetic Data
Source: Genomics Proteomics Bioinformatics. 2019 Nov 23;17(5):503–10. doi: 10.1016/j.gpb.2018.10.006 (PMC7056857; doi:10.1016/j.gpb.2018.10.006)

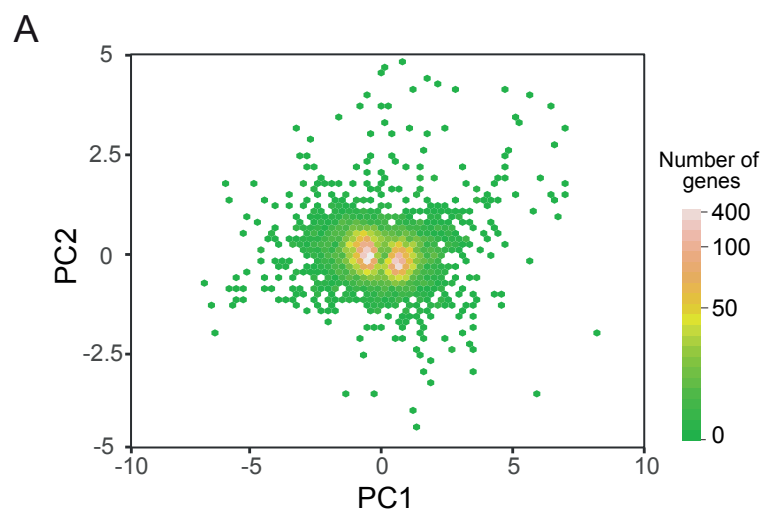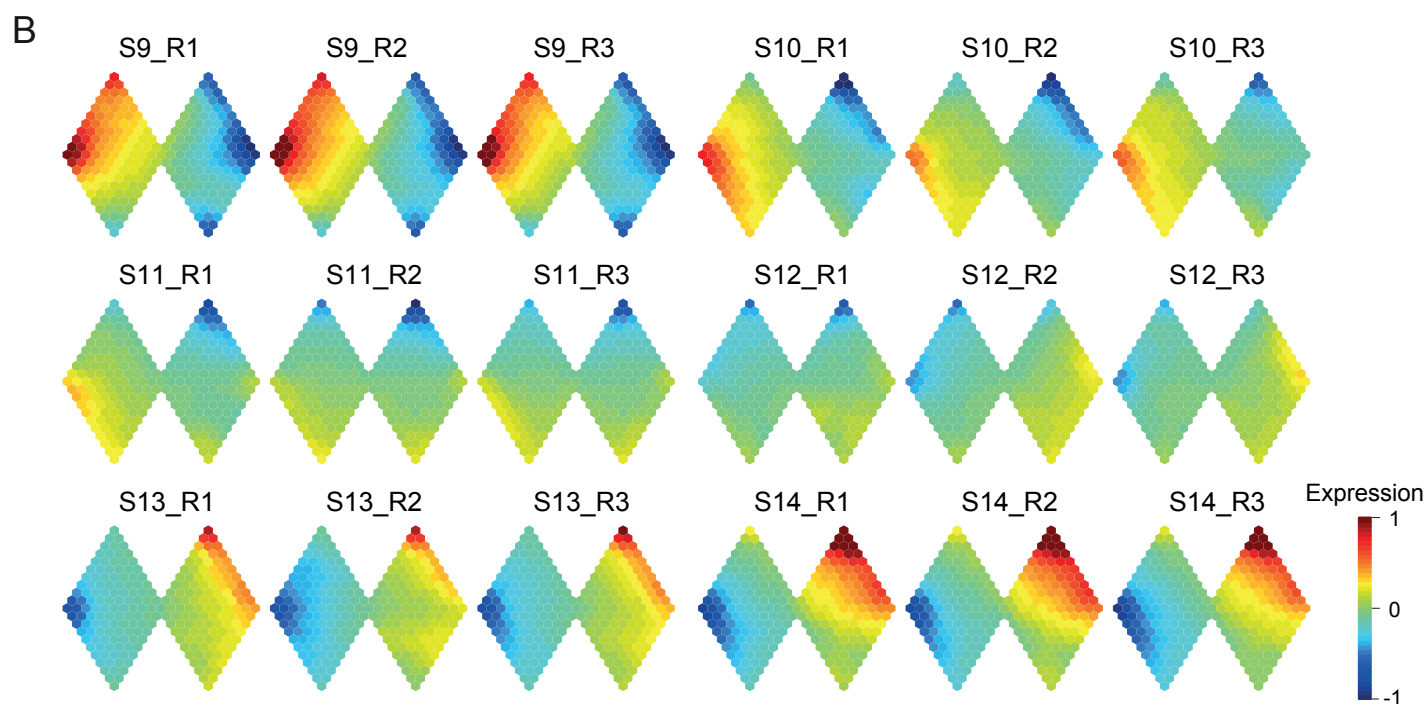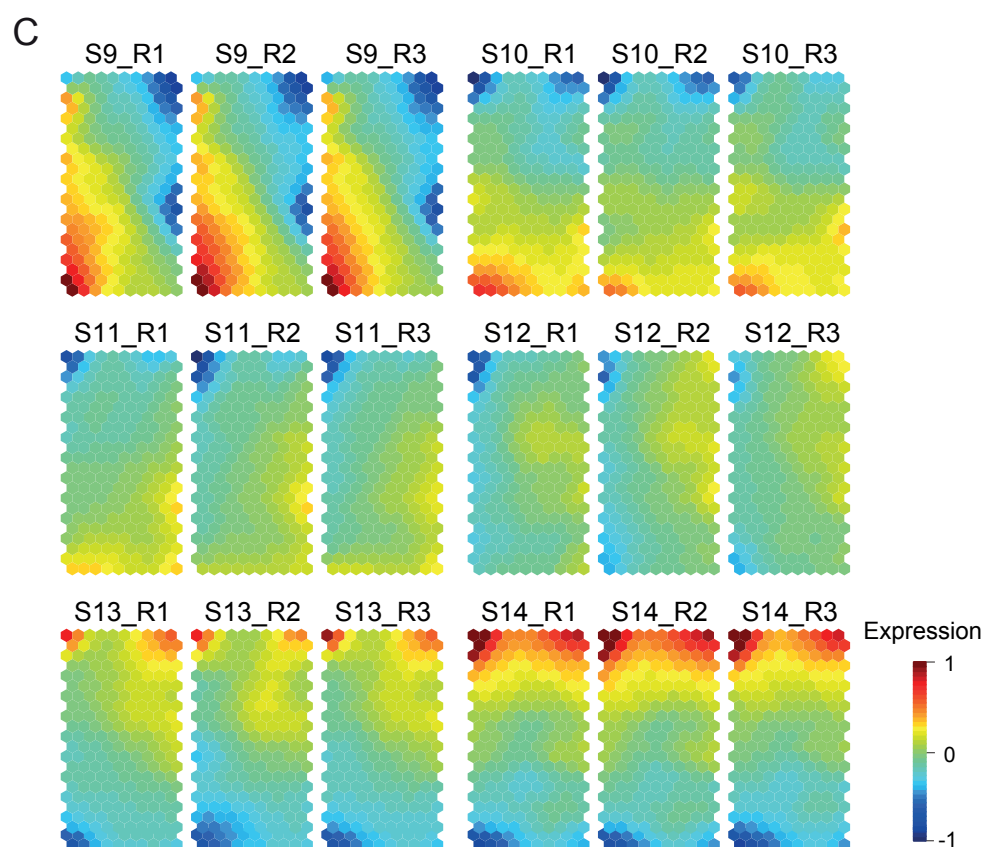

Supplement: Supplementary data 1 [file mmc1.pdf]

A

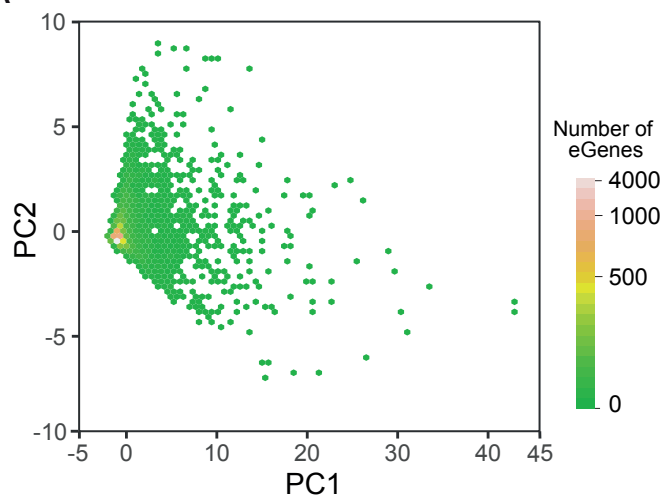

B

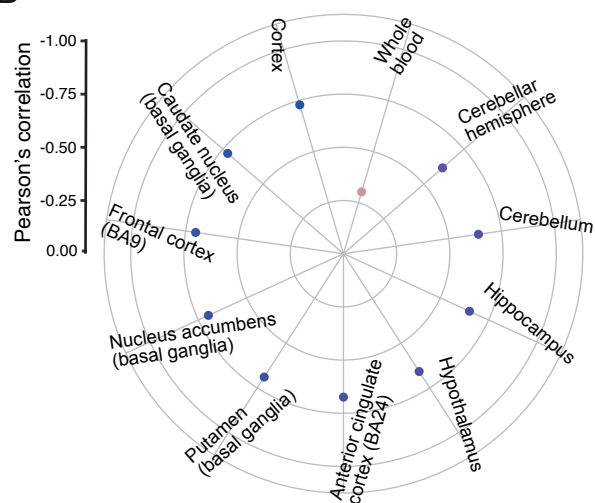

C

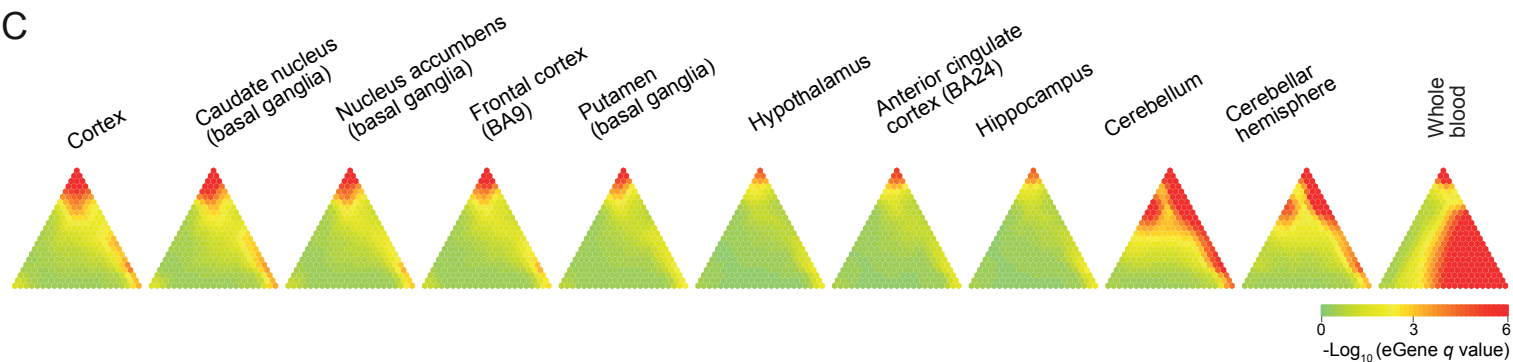

Supplement: Supplementary data 2 [file mmc2.pdf]

A

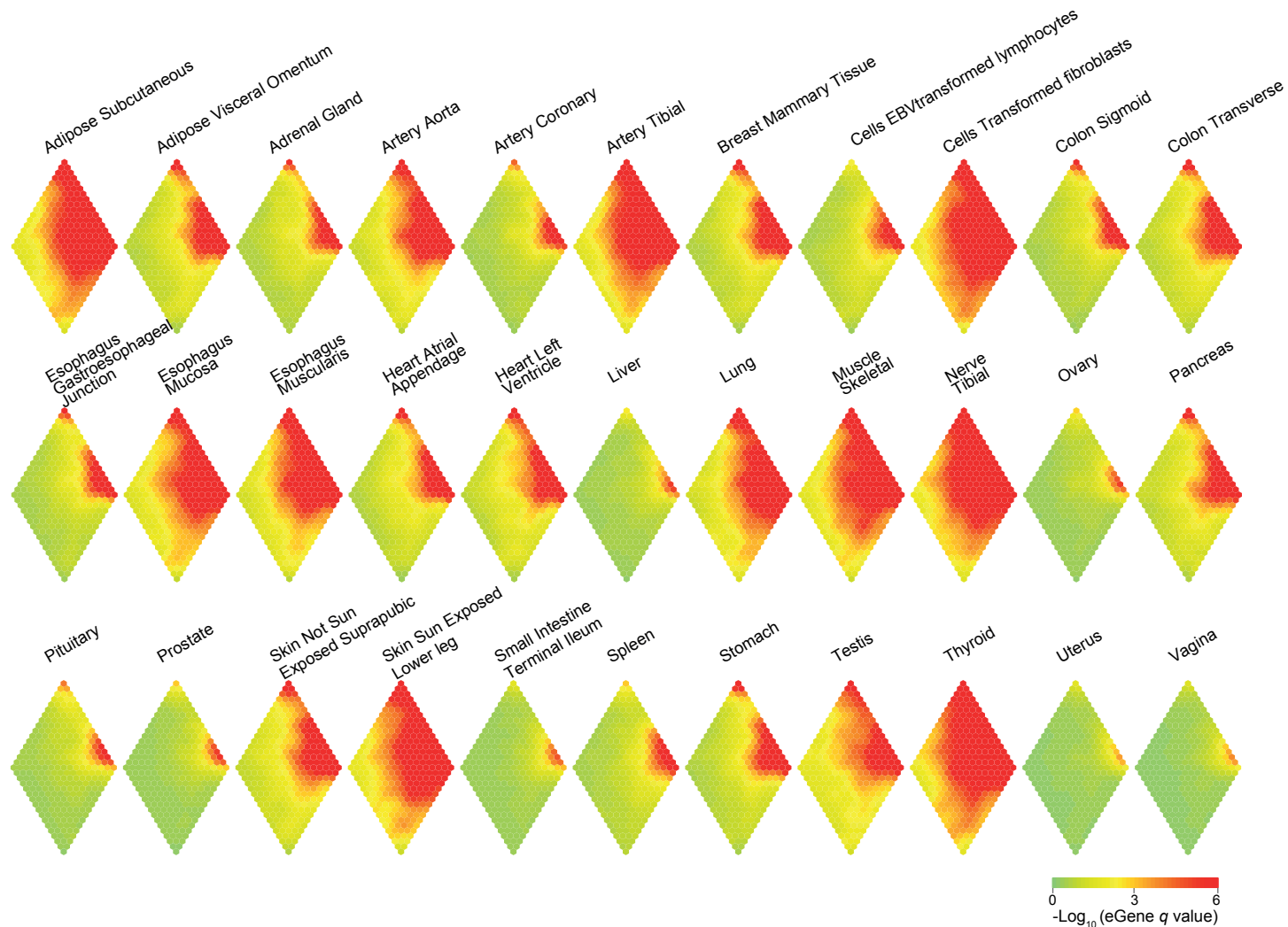

B

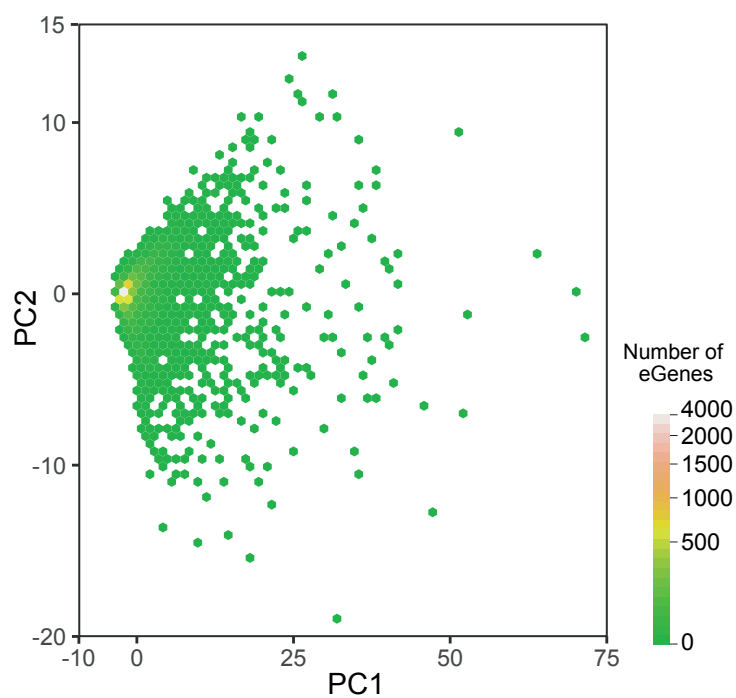

Supplement: Supplementary data 3 [file mmc3.pdf]

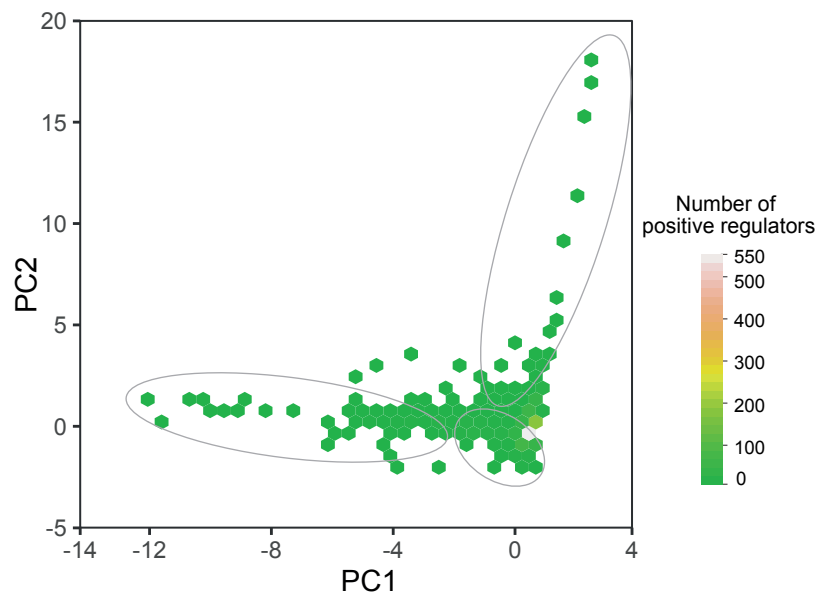

Supplement: Supplementary data 4 [file mmc4.pdf]
